# Supplementary material for: Depletable peroxidase-like activity of Fe3O4 nanozymes accompanied with separate migration of electrons and iron ions
Source: Nat Commun. 2022 Sep 12;13:5365. doi: 10.1038/s41467-022-33098-y (PMC9467987; doi:10.1038/s41467-022-33098-y)
Supplement: Supplementary file 1 — Supplementary Information [file 41467_2022_33098_MOESM1_ESM.pdf]

Supplementary Information

Depletable Peroxidase-like Activity of Fe_3O_4 Nanozymes Accompanied with Separate Migration of Electrons and Iron Ions

Haijiao Dong[1,2], Wei Du[1,2], Jian Dong[1], Renchao Che[3,4,5], Fei Kong[1,2], Wenlong Cheng[6,7], Ming Ma[1,2]*, Ning Gu[1,2]* and Yu Zhang[1,2]*

[1] *State Key Laboratory of Bioelectronics, School of Biological Science and Medical Engineering, Southeast University, Nanjing 210096, P. R. China*

[2] *Jiangsu Key Laboratory for Biomaterials and Devices, School of Biological Science and Medical Engineering, Southeast University, Nanjing 210096, P. R. China*

[3] *Laboratory of Advanced Materials, Fudan University, Shanghai 200438, P. R. China*

[4] *Shanghai Key Lab of Molecular Catalysis and Innovative Materials, Fudan University, Shanghai 200438, P. R. China*

[5] *Department of Materials Science, Fudan University, Shanghai 200438, P. R. China*

[6] *Department of Chemical Engineering, Faculty of Engineering, Monash University, Clayton, Victoria, Australia.*

[7] *The Melbourne Centre for Nanofabrication, Clayton, Victoria, Australia.*

E-mail: maming@seu.edu.cn; guning@seu.edu.cn; zhangyu@seu.edu.cn

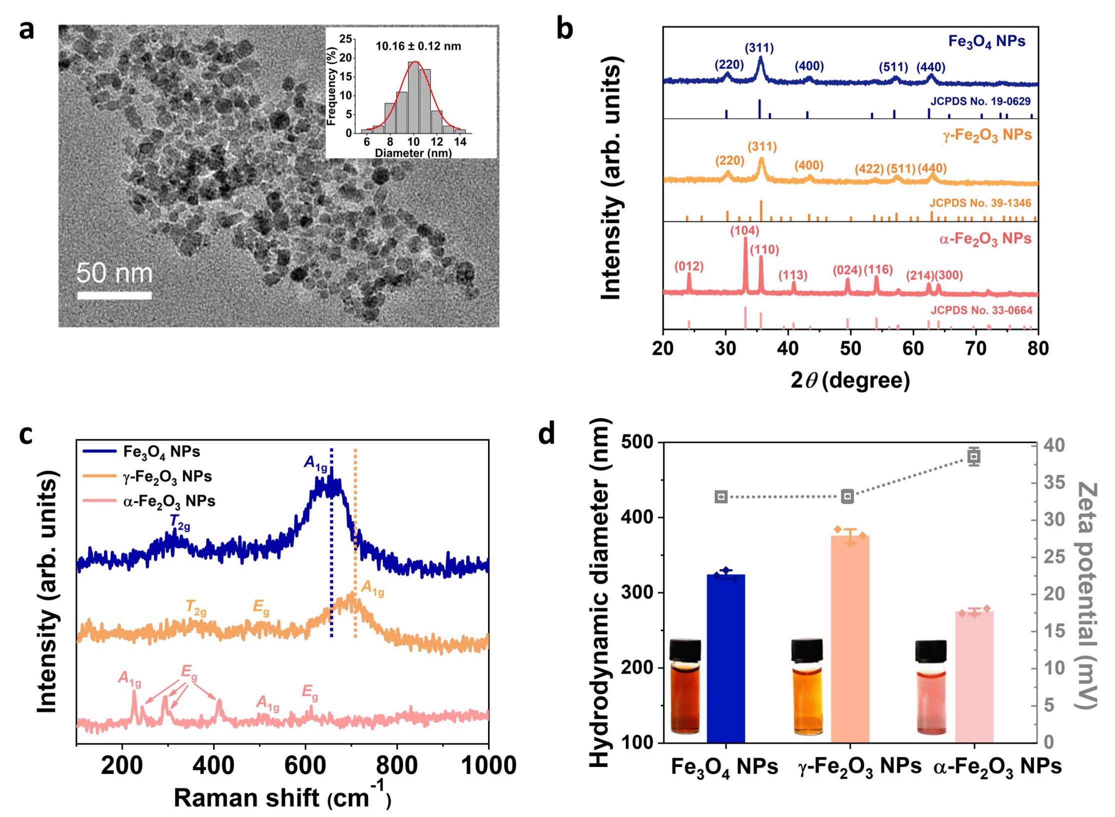

Supplementary Fig. 1 (a) TEM image and size distribution (inset) of Fe_3O_4 NPs synthesized by chemical co-precipitation method. The image was collected at least three times. (b) XRD patterns of as-synthesized Fe_3O_4 NPs, γ-Fe_2O_3 NPs and α-Fe_2O_3 NPs in comparison with the standard diffraction peaks. (c) Ramam spectra of as-synthesized IONPs. (d) Hydrodynamic diameter and Zeta potential of these three IONPs in aqueous solution of pH 3. Error bars represent standard deviation from three independent measurements. Inset: the digital photographs of the three IONPs aqueous solution.

Supplementary Discussion for Supplementary Fig. 1: The XRD patterns of these three iron oxide NPs (IONPs) were in agreement with the corresponding standard diffraction peaks, indicating the acceptable purity and crystallinity (Supplementary Fig. 1b). From the Raman spectra, Fe_3O_4 NPs and γ-Fe_2O_3 NPs were further identified by the A_{1g} mode band near 660 and 700 cm^{-1}, respectively (Supplementary Fig. 1c).[1-5] Based on the literature, magnetite (Fe_3O_4) has three dominant Raman bands at 313 (T_{2g} mode), 542 (T_{2g} mode), 660 (A_{1g} mode) cm^{-1}; mghemite (γ-Fe_2O_3) can be characterized by three broad structures around 360 (T_{2g}), 504 (E_g mode) and 700 cm^{-1} (A_{1g} mode); hematite (α-Fe_2O_3) belongs to the D^6_{3d} crystal space

group and seven phonon lines are expected in the Raman spectrum, namely two A_{1g} modes (229 and 503 cm^{-1}) and five E_g modes (247, 295, 299, 412 and 612 cm^{-1}).[3-5] The hydrodynamic diameters of three IONPs increased because of the slight aggregation of NPs caused by the relatively weak surface stabilization of $N(CH_3)^{4+}$ on the particle surface (Supplementary Fig. 1d).

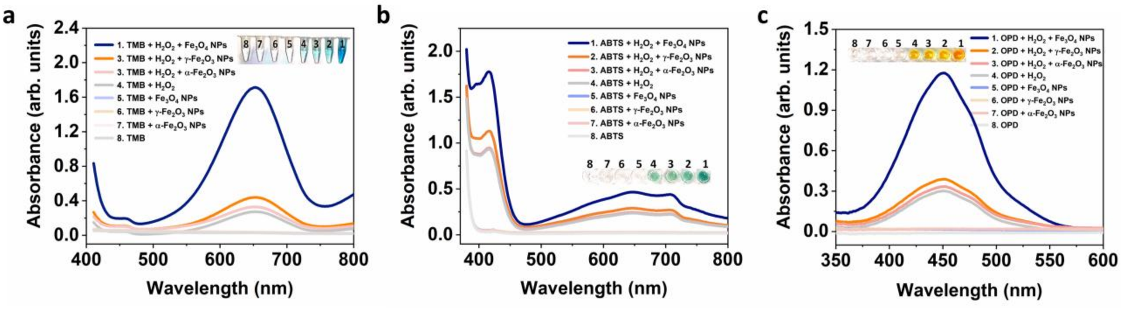

Supplementary Fig. 2 The POD-like activity of as-synthesized Fe_3O_4 NPs, γ-Fe_2O_3 NPs, and α-Fe_2O_3 NPs with (a) TMB, (b) ABTS, and (c) OPD as colorimetric substrates under the presence of H_2O_2 in 0.2 M acetate buffer (pH = 3.6).

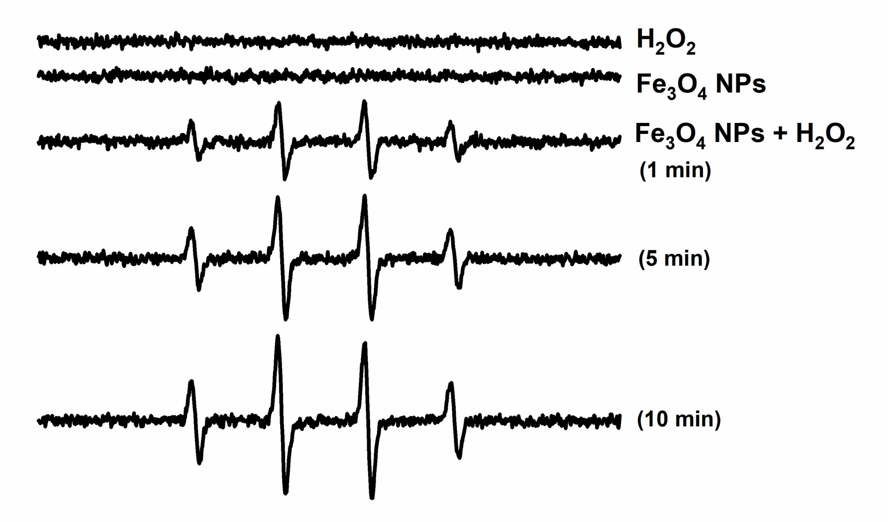

Supplementary Fig. 3 ESR spectra of spin adducts DMPO/·OH produced by Fe_3O_4 NPs (10 μg/mL) in the presence or absence of H_2O_2 (0.165 M) in 0.2 M acetate buffer (pH = 3.6).

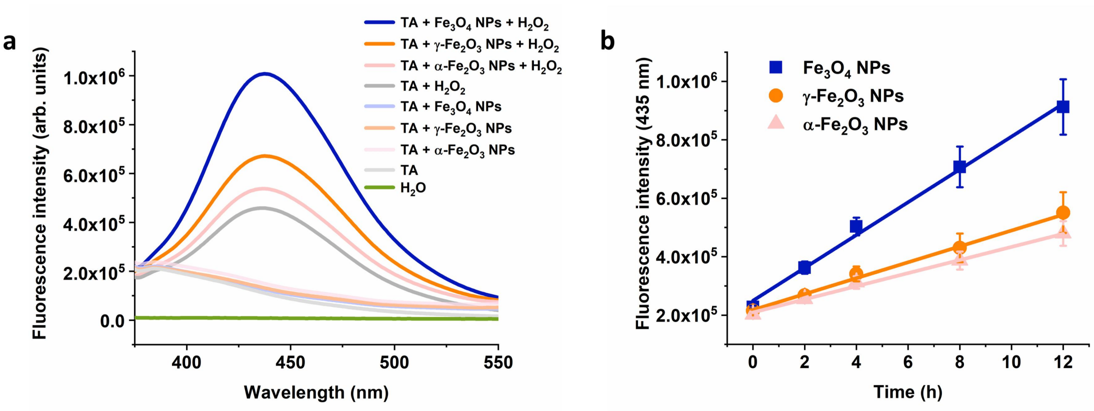

Supplementary Fig. 4 (a) Reaction between terephthalic acid (TA) and ·OH generated by IONPs in the presence and absence of H_2O_2 in 0.2 M acetate buffer (pH = 3.6) after 12 h. (b) Plot of the fluorescence intensity of 2-hydroxyterephthalic acid (TAOH) at 435 nm with reaction time catalyzed by different IONPs. The concentrations of TA, IONPs, and H_2O_2 were 0.5 mM, 4.8 μg/mL, and 0.2 M, respectively. Error bars represent standard deviation from three independent measurements.

a

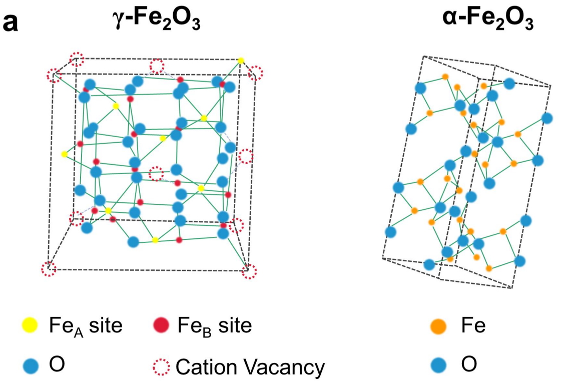

b

$$Fe^{3+} + H_2O_2 \rightarrow FeOOH^{2+} + H^+ \qquad (S1)$$

$$FeOOH^{2+} \rightarrow Fe^{2+} + HO_2 \cdot \qquad (S2)$$

$$Fe^{2+} + H_2O_2 \rightarrow Fe^{3+} + OH^- + \cdot OH \qquad (S3)$$

$$\cdot OH + TMB_{(colourless)} \rightarrow TMB_{ox(blue)} \qquad (S4)$$

Supplementary Fig. 5 (a) Diagram of the crystal structure of γ-Fe_2O_3 and α-Fe_2O_3. (b) Reaction equations for the POD-like activity of IONPs. (Reproduced from Ref. 6 with permission from the Royal Society of Chemistry.)

Supplementary Discussion for Supplementary Fig. 5: As shown in Supplementary Fig. 5a, γ-Fe_2O_3 has a cubic crystal structure of inverse spinel type with cation vacancies in octahedral positions. Most of these cation vacancies are located on the particle surface.[6] As a result, the anionic sites of O on the surface are exposed, allowing substrate H_2O_2 to be easily adsorbed. The adsorbed H_2O_2 undergoes acid-like dissociative chemisorption, producing H^+ and OOH^-. Then, OOH^- reacts with Fe^{3+} to form $FeOOH^{2+}$ (Eq. S1 in Supplementary Fig. 5b). Subsequently, the OOH^- in $FeOOH^{2+}$ donates an electron to Fe^{3+} to form Fe^{2+} (Eq. S2 in Supplementary Fig. 5b), which triggers the Fenton-like reaction (Eq. S3 in Supplementary Fig. 5b), resulting in a relatively higher POD-like activity of γ-Fe_2O_3 NPs (Eq. S4 in Supplementary Fig. 5b). However, these cation vacancies are not existing on the surface of α-Fe_2O_3[6] due to the change of the inverse spinel structure caused by the high calcination temperature (650 °C), leading to a lower affinity to H_2O_2 and the negligible POD-like activity of α-Fe_2O_3.

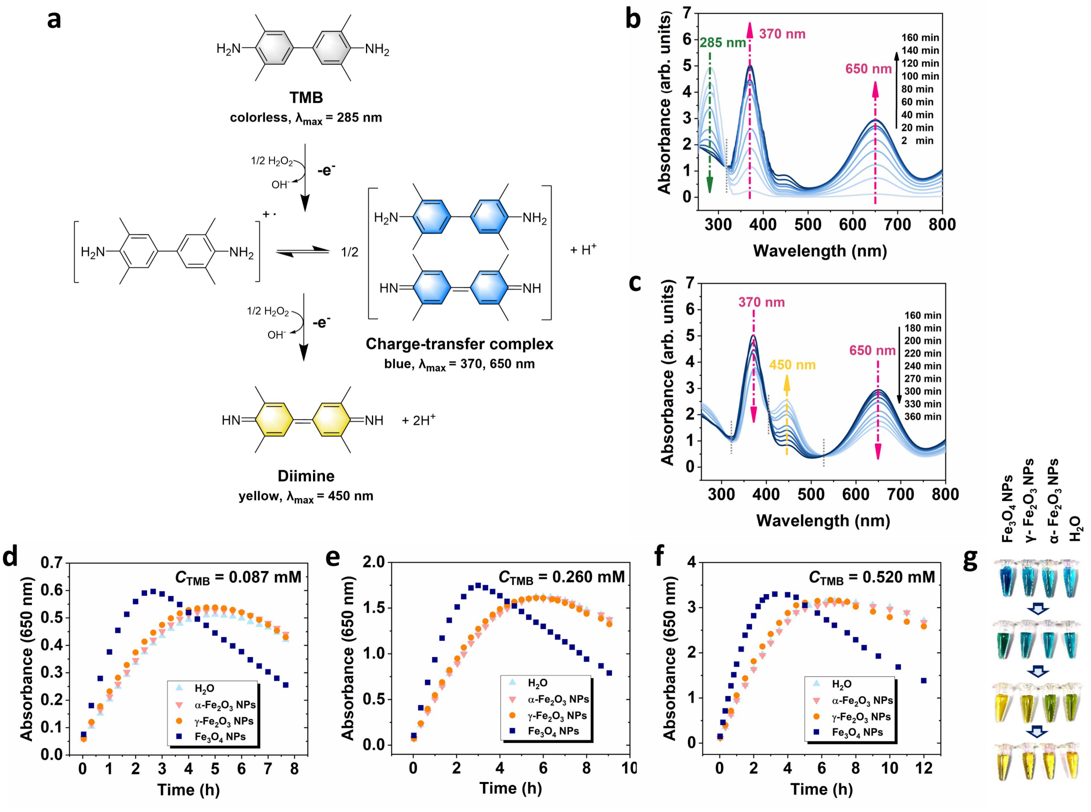

Supplementary Fig. 6 (a) Reaction scheme for the oxidation of TMB. (b and c) The changes of UV-Vis spectra during the oxidation of TMB catalyzed by Fe_3O_4 NPs in the presence of H_2O_2 in 0.2 M acetate buffer (pH = 3.6). (d-f) The absorbance changes at 650 nm catalyzed by Fe_3O_4 NPs, γ-Fe_2O_3 NPs, and α-Fe_2O_3 NPs (0.625 μg Fe/mL) with different concentration of TMB in the presence of H_2O_2 (7 mM) at pH 3.6. (g) The color changes of POD-like reaction solution catalyzed by Fe_3O_4 NPs, γ-Fe_2O_3 NPs, and α-Fe_2O_3 NPs.

Supplementary Discussion for Supplementary Fig. 6: The oxidation of TMB by peroxidase involves two successive one-electron transfer processes (Supplementary Fig. 6a).[7] At the beginning of oxidation reaction, colorless TMB loses one electron to form the blue charge-transfer complex with maximum absorbance peaks at 370 and 650 nm (Supplementary Fig. 6b). In the second stage, this blue complex continues to lose an electron, forming the final yellow diimine with maximum absorbance peaks of 450 nm (Supplementary Fig. 6c). Since the peak at 370 nm overlaps with the diimine peak at 450 nm, the absorption at 650 nm was used to follow the kinetics of catalytic reaction (Supplementary Figs. 6d-f).

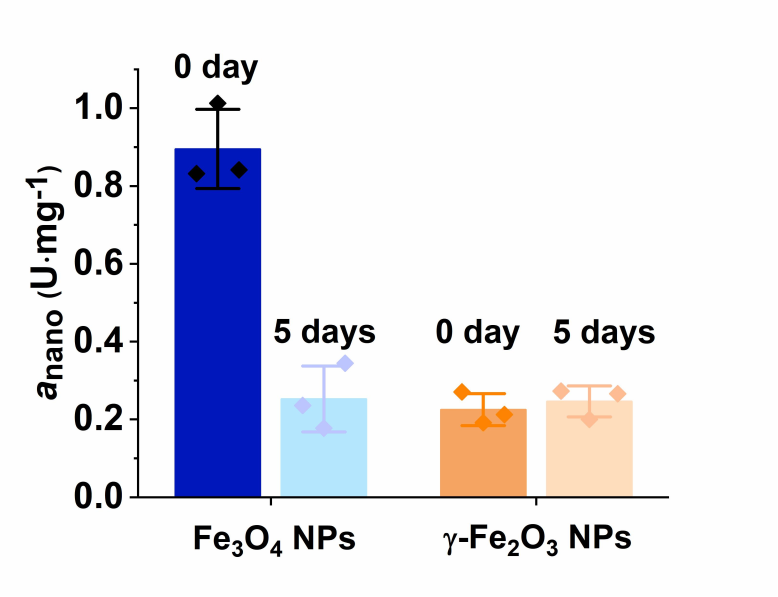

Supplementary Fig. 7 The a_{nano} of Fe_3O_4 NPs and γ-Fe_2O_3 NPs before and after 5 days of cyclic catalysis. Error bars represent standard deviation from three independent measurements.

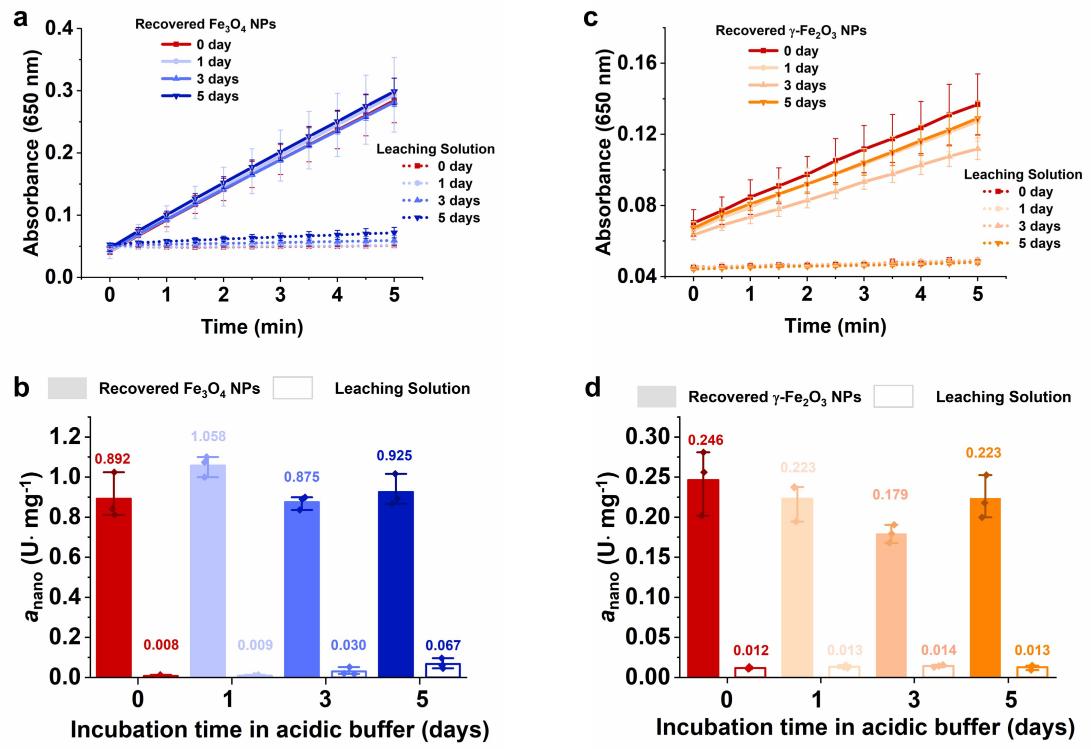

Supplementary Fig. 8 The POD-like activity assessment of the recovered NPs and leaching solution after different incubation times in acidic buffer solution (pH=3.6). (a and b) Fe_3O_4 NPs; (c and d) γ-Fe_2O_3 NPs. Error bars represent standard deviation from three independent measurements.

Supplementary Discussion for Supplementary Fig. 8: In acidic medium, Fe ions may release from the IONPs. Thus it is important to exclude the possible influence of Fe ions leaching effect on the catalytic activity of the recycled IONPs after participating in cyclic catalysis. To test this, we incubated the Fe_3O_4 NPs or γ-Fe_2O_3 NPs in acetate buffer (pH 3.6) and compared the POD-like activity of the leaching solution with that of recovered NPs on days 0, 1, 3 and 5. As shown in Supplementary Fig. 8, the leaching solution within 5 days of incubation showed marginal catalytic activity. Besides, the POD-like activity of the recovered NPs did not show a significantly negative correlation with the incubation time. We also measured the Fe content in the leaching solution using ICP-MS (Supplementary Table 1), which is 1-2 orders of magnitude less than the concentration needed for the Fenton reaction. These indicate that the leached Fe ions does not contribute significantly to the reduction of the catalytic ability of Fe_3O_4 NPs after cyclic catalysis.

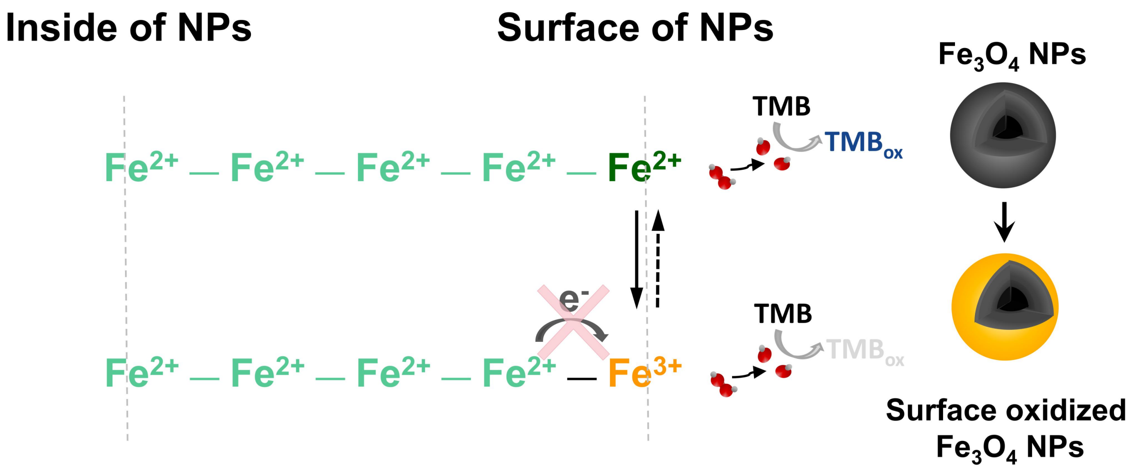

Supplementary Fig. 9 Only the surface active sites (Fe^{2+}) of Fe_3O_4 nanozymes involve in the POD-like catalytic reaction.

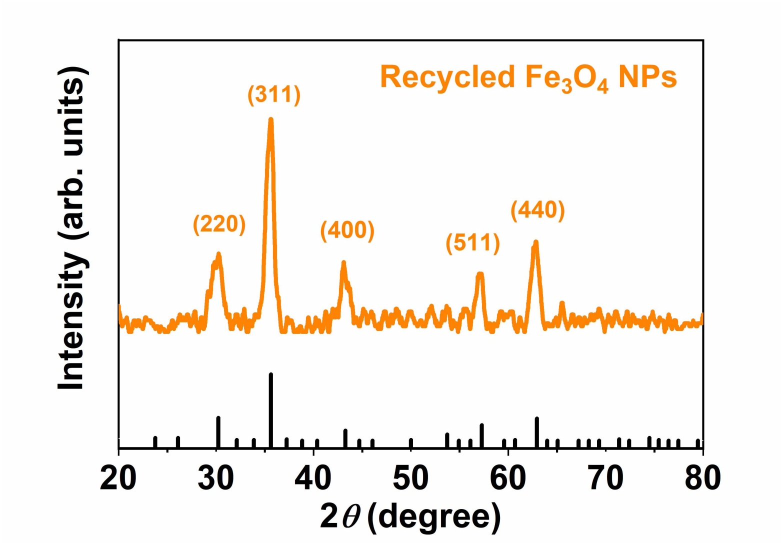

Supplementary Fig. 10 XRD pattern of the recycled Fe_3O_4 NPs after 5 days of cyclic catalysis. Black lines represent the standard pattern of γ-Fe_2O_3.

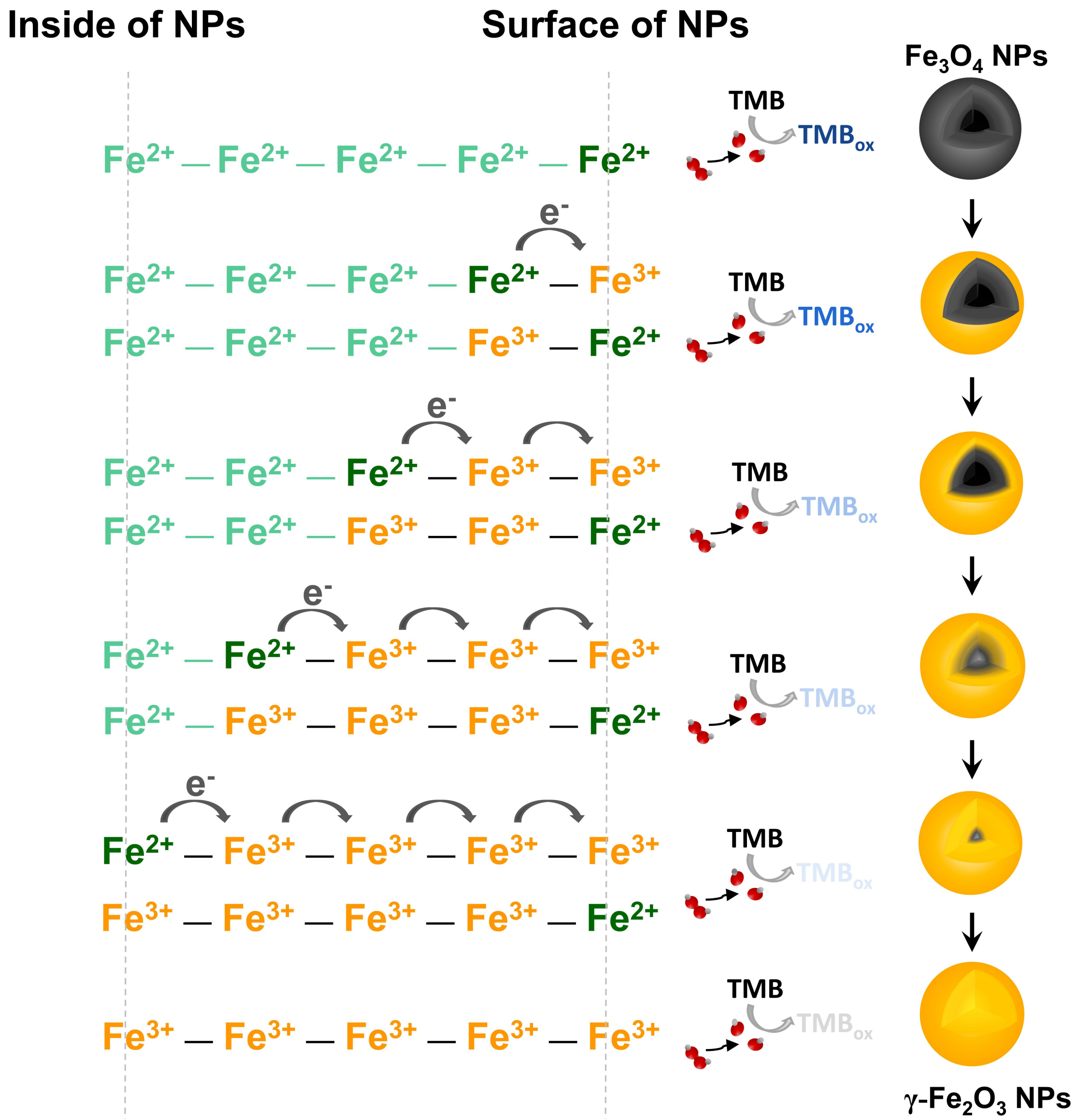

Supplementary Fig. 11 The inside Fe^{2+} transfer electron to particle surface to maintain the POD-like catalytic capacity of Fe_3O_4 nanozymes.

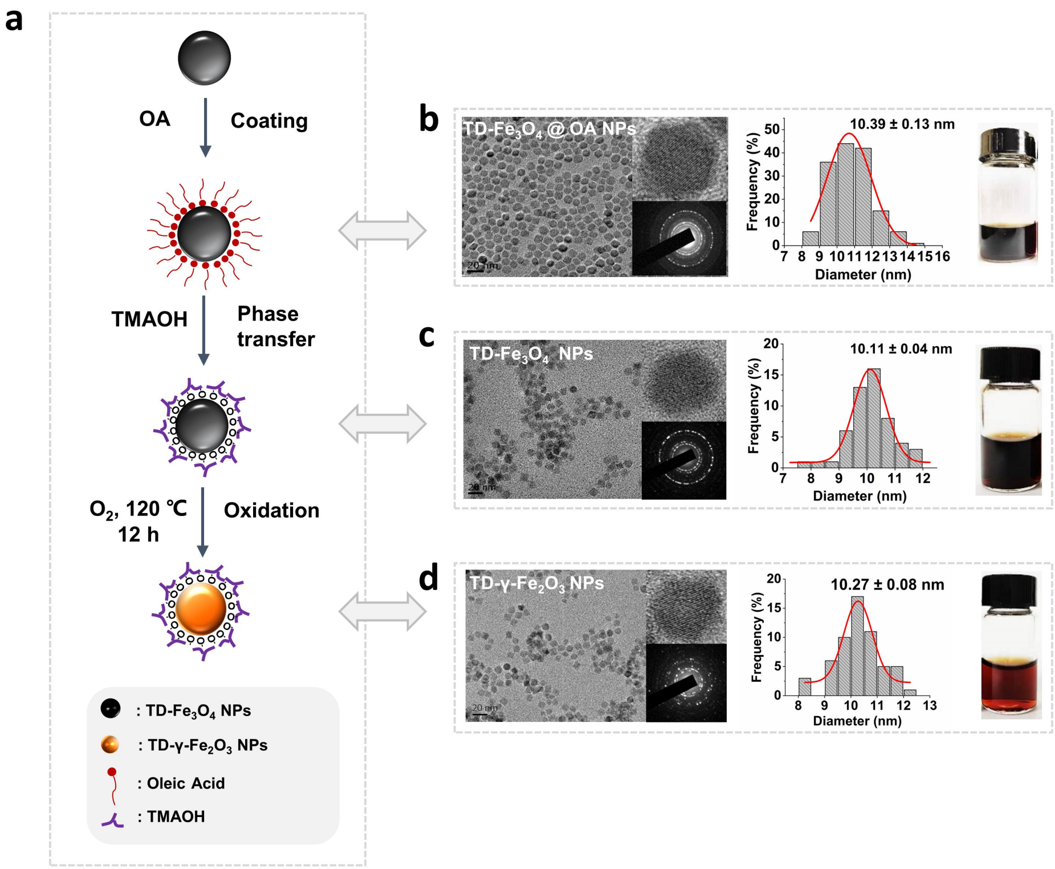

Supplementary Fig. 12 (a) Illustration of the synthesis process of Fe_3O_4 NPs by thermal decomposition methods (named TD-Fe_3O_4 NPs) and the aeration oxidization of TD-Fe_3O_4 NPs. (b-d) TEM image, size distribution, and digital photo of NPs at each synthesis stage. Inset: HRTEM and corresponding SAED pattern. Images were collected at least three times for each type of NPs.

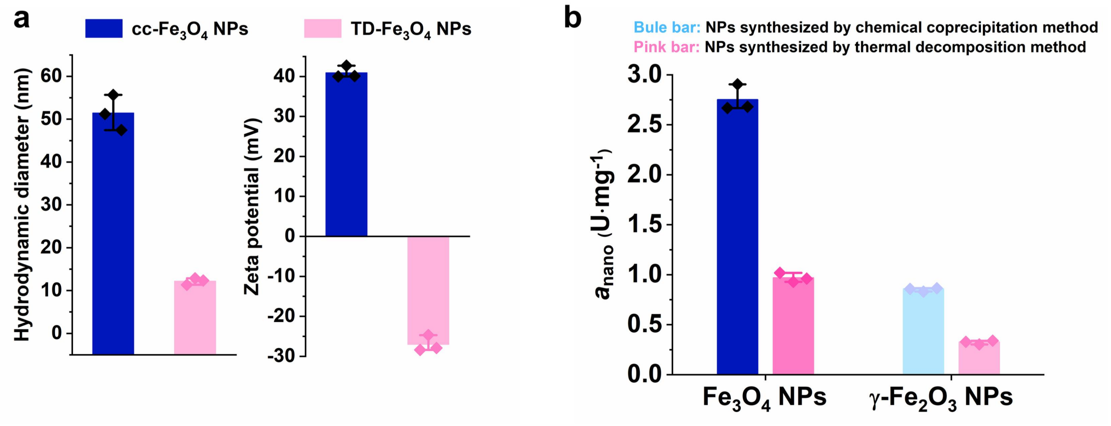

Supplementary Fig. 13 (a) Comparison of the hydrodynamic diameter and Zeta potential of cc-Fe_3O_4 NPs and TD-Fe_3O_4 NPs. (b) Comparison of a_{nano} of Fe_3O_4 NPs synthesized by two methods before and after aeration oxidation of 12 h. Error bars represent standard deviation from three independent measurements.

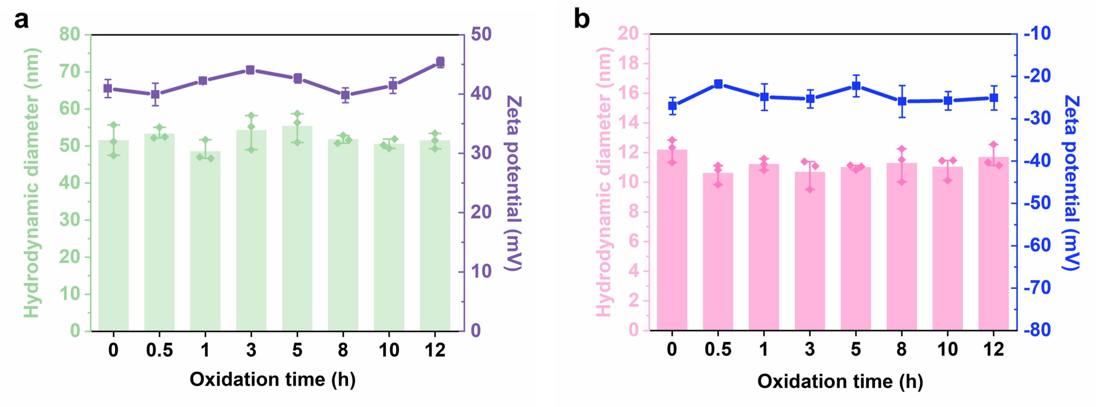

Supplementary Fig. 14 Changes in hydrodynamic diameter and Zeta potential of (a) cc-Fe_3O_4 NPs and (b) TD-Fe_3O_4 NPs during the aeration oxidation. Error bars represent standard deviation from three independent measurements.

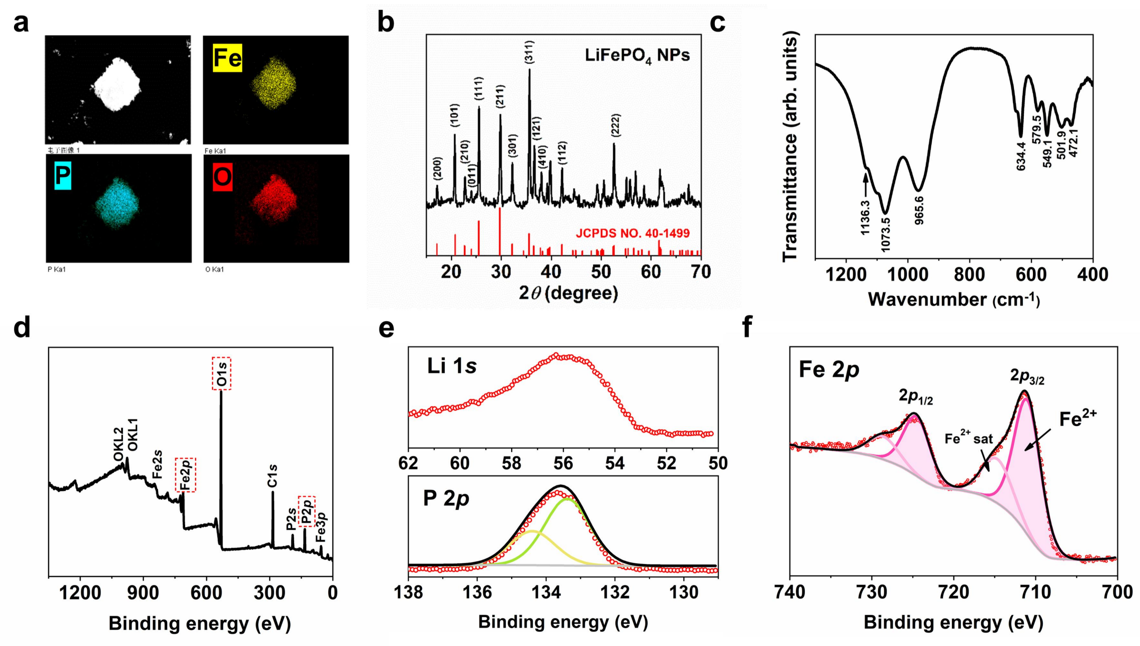

Supplementary Fig. 15 The characterization of $LiFePO_4$ NPs. (a) The element mapping of as-synthesized $LiFePO_4$ NPs. (b) XRD, (c) FTIR and (d) XPS spectra of as-synthesized $LiFePO_4$ NPs. The core XPS spectra of (e) Li 1*s*, P 2*p*, and (f) Fe 2*p* of as-synthesized $LiFePO_4$ NPs.

Supplementary Discussion for Supplementary Fig. 15: Element mapping of the $LiFePO_4$ powders indicate the presence of Fe, P, and O elements in the prepared samples (Supplementary Fig. 15a). The sharp XRD diffraction peak, shown in Supplementary Fig. 15b, is identified as the orthorhombic olivine structure (JCPDS NO. 40-1499) of $LiFePO_4$, suggesting the phase purity and the high crystallinity of the samples.[8] Additionally, the FTIR spectrum in the ranges of 1150-950 cm^{-1} and 650-450 cm^{-1} are characteristic for the absorption of PO_4^{3-} in $LiFePO_4$ NPs (Supplementary Fig. 15c), and their corresponding vibration models were summarized in Supplementary Table 2, agreeing well with the results reported in the literature.[9,10] The XPS full spectrum of as-synthesized $LiFePO_4$ NPs and the core spectra in the binding energy range of Li 1*s*, P 2*p*, and Fe 2*p* were shown in Supplementary Figs. 15d-f. Detailed information was listed in Supplementary Table 3. Notably, the Fe 2*p* spectrum contains two main peaks (710.98 and 724.50 eV) and their satellite peak at corresponding high binding energies position. The spin-orbital split energy is about 13.5 eV. These imply that the chemical oxidation state of Fe in the as-synthesized

$LiFePO_4$ NPs is divalent.[8,11,12] Furthermore, the ICP analysis shows that the molar ratio of Li: Fe: P in the prepared $LiFePO_4$ is 1: 1.04: 1.02, which approximately matched its stoichiometric formula.[13] The above characterization results indicate that $LiFePO_4$ NPs have been successfully synthesized.

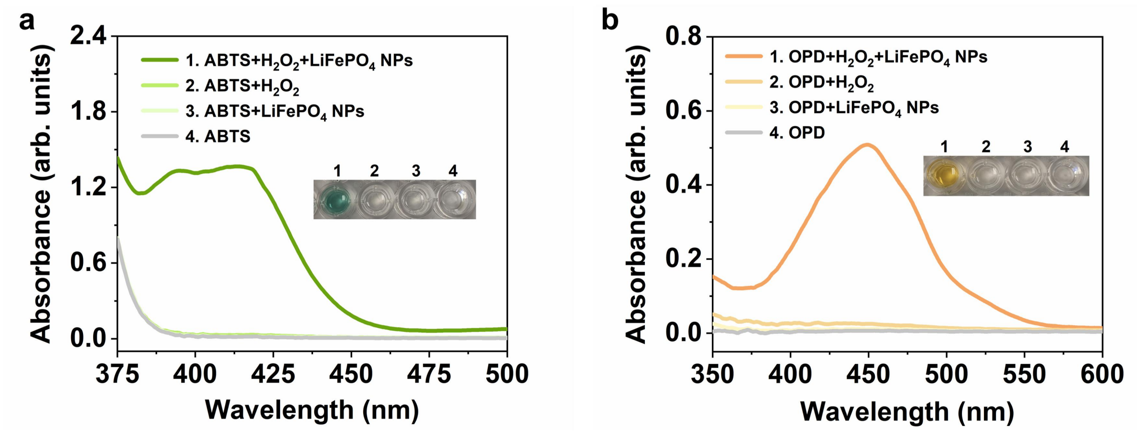

Supplementary Fig. 16 The POD-like activity of $LiFePO_4$ NPs with (a) ABTS and (b) OPD as colorimetric substrates in the presence of H_2O_2 at pH 3.6.

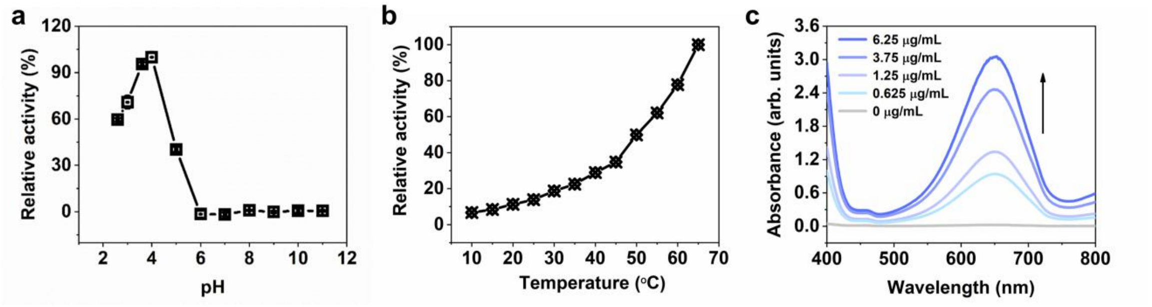

Supplementary Fig. 17 (a) pH, (b) temperature, and (c) NPs concentration-dependence of the POD-like activity of $LiFePO_4$ NPs.

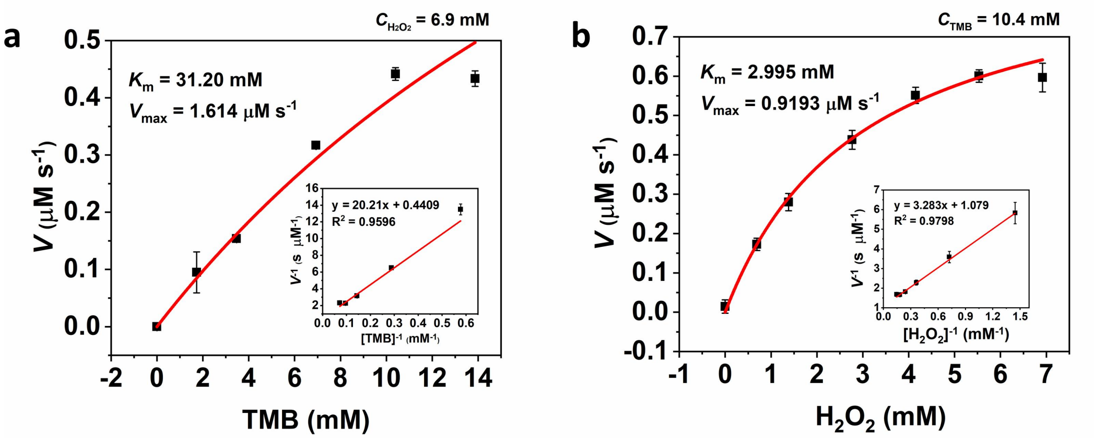

Supplementary Fig. 18 Steady-state kinetic assay of $LiFePO_4$ NPs with (a) TMB and (b) H_2O_2 as substrates. Insets are the Lineweaver-Burk fitting. Error bars represent standard deviation from three independent measurements.

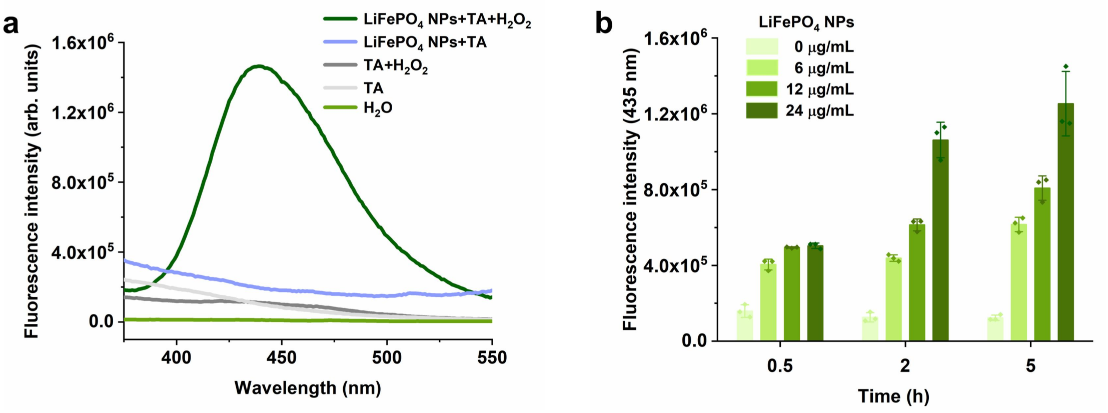

Supplementary Fig. 19 (a) Reaction between terephthalic acid (TA) and ·OH generated by $LiFePO_4$ NPs in the presence and absence of H_2O_2 in 0.2 M acetate buffer (pH = 3.6) after 5 h. The concentrations of TA, $LiFePO_4$ NPs, and H_2O_2 were 0.5 mM, 24 μg/mL, and 0.2 M, respectively. (b) Plot of the fluorescence intensity of 2-hydroxyterephthalic acid (TAOH) at 435 nm against the reaction time and concentration of $LiFePO_4$ NPs. Error bars represent standard deviation from three independent measurements.

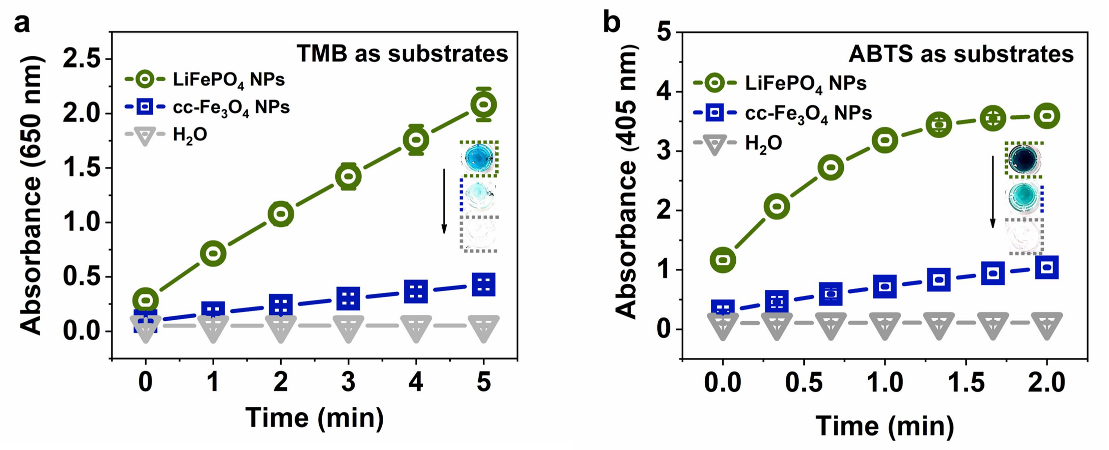

Supplementary Fig. 20 The POD-like activity of $LiFePO_4$ NPs and cc-Fe_3O_4 NPs at the same concentration (0.4 μg Fe/mL) for (a) TMB (1.7 mM) and (b) ABTS (0.75 mM) coloration in the presence of H_2O_2 (0.8 M) at pH 3.6. Error bars represent standard deviation from three independent measurements.

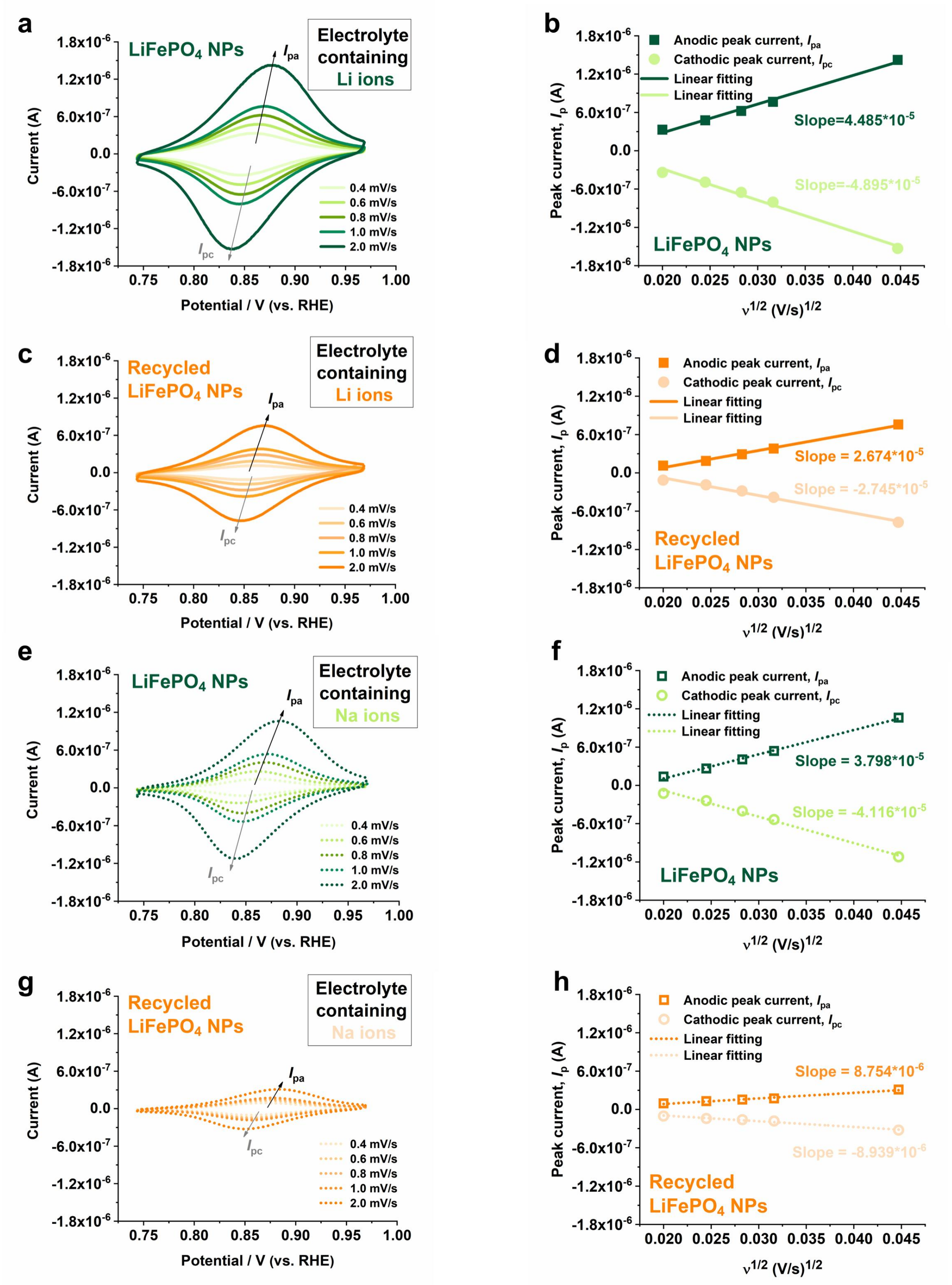

Supplementary Fig. 21 Cyclic voltammograms of (a and e) $LiFePO_4$ NPs and (c and g) recycled $LiFePO_4$ NPs at various scan rates of 0.4~2.0 mV s^{-1}. Plot of peak currents (i_{pa} and i_{pc}) vs. square root of scan rates ($v^{1/2}$) of (b and f) $LiFePO_4$ NPs and (d and h) recycled $LiFePO_4$ NPs. The electrolytes used for the experiments in Figs. a-d and Figs. e-h are lithium acetate buffers and sodium acetate buffer, respectively.

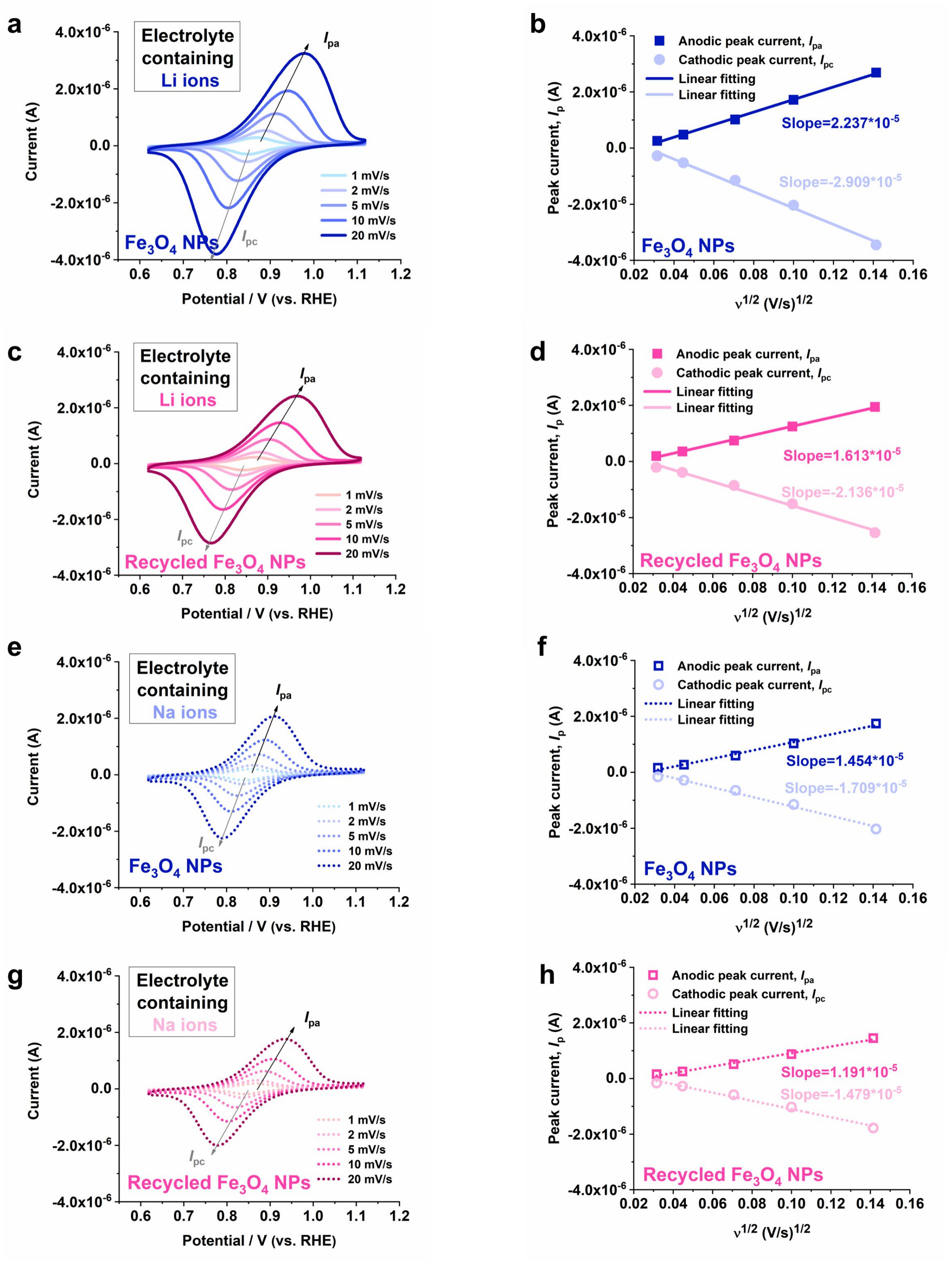

Supplementary Fig. 22 Cyclic voltammograms of (a and e) Fe_3O_4 NPs and (c and g) recycled Fe_3O_4 NPs at various scan rates of 1~20 mV s^{-1}. Plot of peak currents (i_{pa} and i_{pc}) vs. square root of scan rates ($v^{1/2}$) of (b and f) Fe_3O_4 NPs and (d and h) recycled Fe_3O_4 NPs. The electrolytes used for the experiments in Figs. a-d and Figs. e-h are lithium acetate buffers and sodium acetate buffer, respectively.

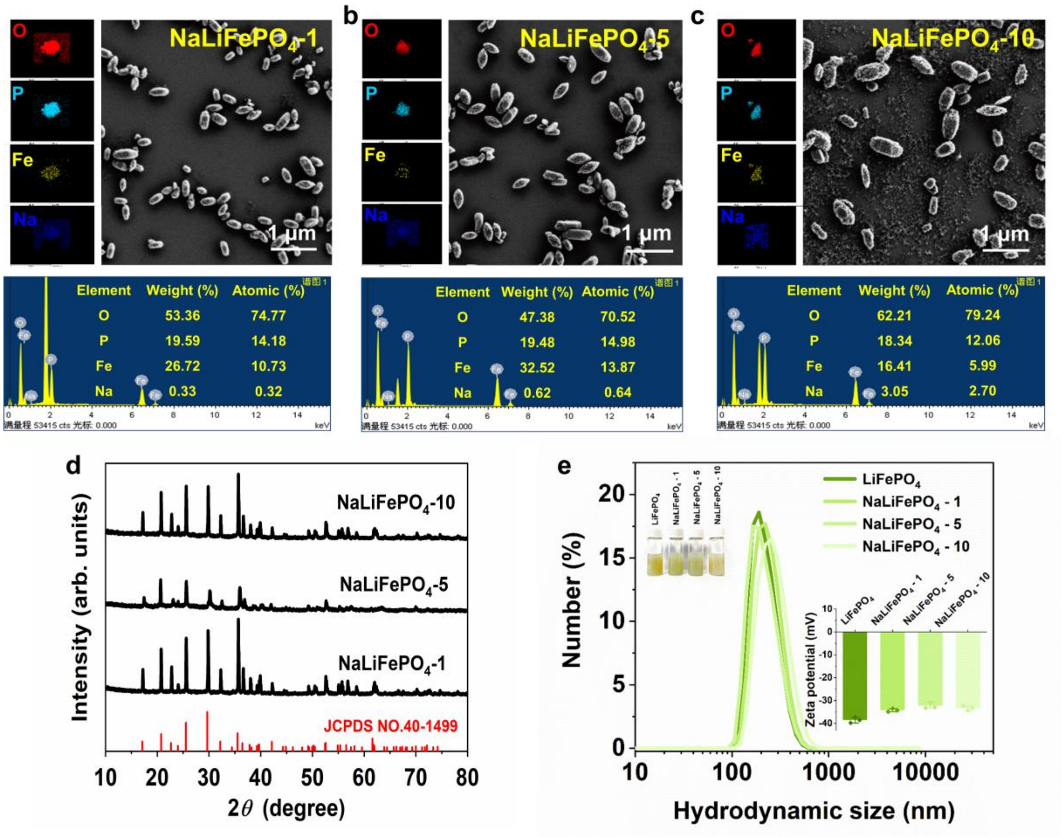

Supplementary Fig. 23 (a-c) SEM image, element mapping, and EDS analysis, (d) XRD patterns, and (e) hydrodynamic diameter and (inset) Zeta potential of NaLiFePO4-1, NaLiFePO4-5, and NaLiFePO4-10. The SEM images were collected at least three times for each type of NPs. Error bars represent standard deviation from three independent measurements.

Supplementary Discussion for Supplementary Fig. 23: Based on the synthetic strategy of $LiFePO_4$ NPs,[10] Na-doped $LiFePO_4$ NPs with similar physicochemical properties were prepared by replacing 1%, 5%, and 10% of the Li source with Na source, named as NaLiFePO4-1, NaLiFePO4-5, and NaLiFePO4-10, respectively. EDS (Supplementary Figs. 23a-c) and ICP (Supplementary Table 4) results show that the amount of Na and the molar ratio of Na: Li in NPs increased with the feeding amount of Na. In addition, the doping of Na did not affect the original orthorhombic olivine structure of $LiFePO_4$ (Supplementary Fig. 23d). They have an approximate hydrodynamic dimension and surface negative potential, compared with $LiFePO_4$ NPs (Supplementary Fig. 23e).

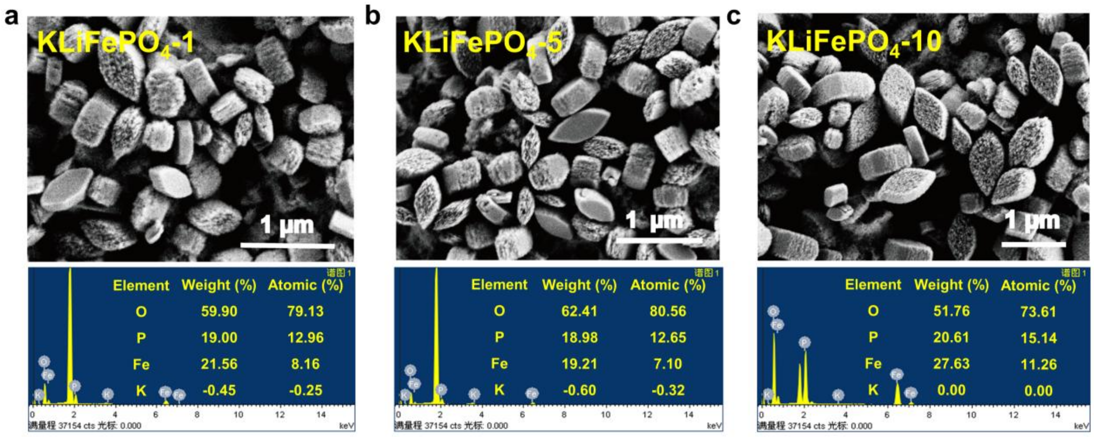

Supplementary Fig. 24 SEM image and EDS analysis of (a) $KLiFePO_4$-1, (b) $KLiFePO_4$-5, and (c) $KLiFePO_4$-10. The images were collected at least three times for each type of NPs.

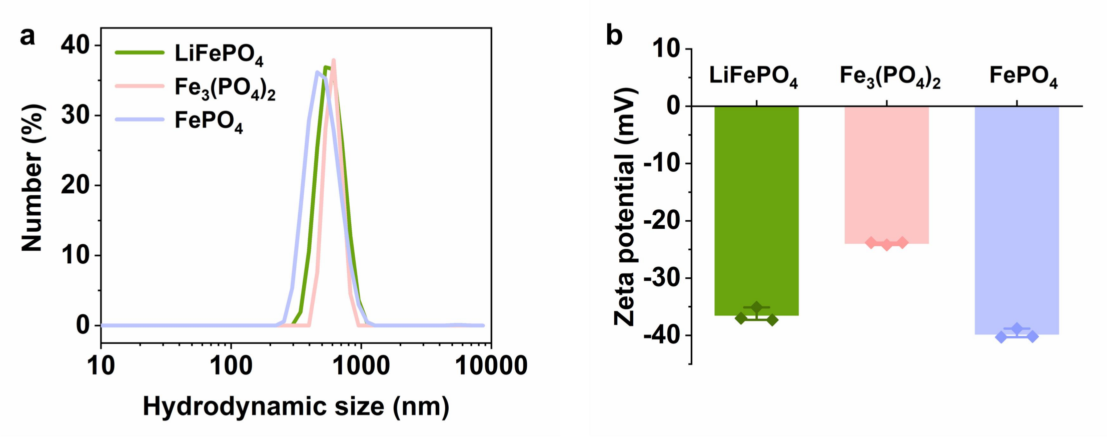

Supplementary Fig. 25 (a) Hydrodynamic diameter and (b) Zeta potential of commercial $LiFePO_4$, $Fe_3(PO_4)_2$, and $FePO_4$ aqueous solution. Error bars represent standard deviation from three independent measurements.

Supplementary Table 1. Leaching Fe ions concentration of Fe_3O_4 NPs or γ-Fe_2O_3 NPs after incubation in acidic buffer solution (pH=3.6).

Incubation time in acidic buffer solution (days)	Leaching Fe ions concentration (μg/mL)	
	Fe_3O_4 NPs	γ-Fe_2O_3 NPs
0	0.007 ± 0.000	
1	0.117 ± 0.026	0.027 ± 0.002
3	0.224 ± 0.006	0.029 ± 0.002
5	0.314 ± 0.008	0.028 ± 0.000

Note: The results are expressed as the mean ± standard deviation of three parallel experiments.

Supplementary Table 2. FTIR spetrum of $LiFePO_4$ NPs.[9,10]

Vibration Model	Wavenumber (cm^{-1})
Stretching mode of O-P-O	1136.3
Anti-symmetric stretching mode of P-O	1073.5
Symmetric stretching mode of P-O	965.6
	634.4
Anti-symmetric bending mode of O-P-O	579.5
symmetric bending mode of O-P-O	549.1
	501.9
Mixture of symmetric and anti-symmetric bending mode of O-P-O	472.1

Supplementary Table 3. XPS binding energy of various atoms for $LiFePO_4$ NPs.[8,11,12]

Atom	Emission peak	Binding energy (eV)	Assignation
Li	1*s*	55.49	Li^{+}
Fe	$2p_{3/2}$	710.98	Fe^{2+}
		714.71	Satellite
	$2p_{1/2}$	724.50	Fe^{2+}
		728.61	Satellite
P	$2p_{3/2}$	133.38	
	$2p_{1/2}$	134.40	$[PO_4]^{3-}$
O	1*s*	531.08	

Supplementary Table 4. ICP analysis results of the Na and K-doped $LiFePO_4$ NPs.

Nanozymes		Molar ratio of Na or K : Li
Na-doped $LiFePO_4$ NPs	$NaLiFePO_4$-1	0.25 : 1
	$NaLiFePO_4$-5	0.80 : 1
	$NaLiFePO_4$-10	1.33 : 1
K-doped $LiFePO_4$ NPs	$KLiFePO_4$-1	0.04 : 1
	$KLiFePO_4$-5	0.05: 1
	$KLiFePO_4$-10	0.04: 1

Supplementary References

1. Zhang, L. et al. Facile synthesis of iron oxides/reduced graphene oxide composites: application for electromagnetic wave absorption at high temperature. *Sci. Rep.-UK*. **5**, 9298 (2015).
2. Gao, J. et al. One-step solvothermal synthesis of highly water-soluble, negatively charged superparamagnetic Fe_3O_4 colloidal nanocrystal clusters. *Nanoscale*. **5**, 7026 (2013).
3. Shebanova, O. N. & Lazor, P. Raman study of magnetite (Fe_3O_4): laser-induced thermal effects and oxidation. *J. Raman Spectrosc.* **34**, 845-852 (2003).
4. Guo, C., Hu, Y., Qian, H., Ning, J. & Xu, S. Magnetite (Fe_3O_4) tetrakaidecahedral microcrystals: synthesis, characterization, and micro-Raman study. *Mater. Charact.* **62**, 148-151 (2011).
5. Jubb, A. M. & Allen, H. C. Vibrational spectroscopic characterization of hematite, maghemite, and magnetite thin films produced by vapor deposition. *ACS Appl. Mater. Inter.* **2**, 2804-2812 (2010).
6. Masud, M. K. et al. Nanoarchitectured peroxidase-mimetic nanozymes: mesoporous nanocrystalline α- or γ-iron oxide? *J. Mater. Chem. B*. **7**, 5412-5422 (2019).
7. Gu, S., Risse, S., Lu, Y. & Ballauff, M. Mechanism of the oxidation of 3,3',5,5'-tetramethylbenzidine catalyzed by peroxidase-like Pt nanoparticles immobilized in spherical polyelectrolyte brushes: a kinetic study. *ChemPhysChem*. **21**, 450-458 (2019).
8. Ma, J., Li, B., Du, H., Xu, C. & Kang, F. Inorganic-based sol-gel synthesis of nano-structured $LiFePO_4$/C composite materials for lithium ion batteries. *J. Solid State Electr.* **16**, 1353-1362 (2012).
9. Salah, A. et al. Local structure and redox energies of lithium phosphates with olivine- and Nasicon-like structures. *J. Power Sources*. **140**, 370-375 (2005).
10. Wang, L. et al. Crystal orientation tuning of $LiFePO_4$ nanoplates for high rate lithium battery cathode materials. *Nano Lett.* **12**, 5632-5636 (2012).
11. Bhuvaneswari, M. S., Bramnik, N. N., Ensling, D., Ehrenberg, H. & Jaegermann, W.

Synthesis and characterization of carbon nano fiber/$LiFePO_4$ composites for Li-ion batteries. *J. Power Sources*. **180**, 553-560 (2008).

12. Caballero, A., Cruz Yusta, M., Morales, J., Santos Peña, J. & Rodríguez Castellón, E. A new and fast synthesis of nanosized $LiFePO_4$ electrode materials. *Eur. J. Inorg. Chem.* **2006**, 1758-1764 (2006).
13. Kim, J. et al. Enhancement of electrochemical performance of lithium iron phosphate by controlled sol-gel synthesis. *Electrochim. Acta*. **53**, 8258-8264 (2008).
