# Supplementary material for: Depletable peroxidase-like activity of Fe3O4 nanozymes accompanied with separate migration of electrons and iron ions
Source: Nat Commun. 2022 Sep 12;13:5365. doi: 10.1038/s41467-022-33098-y (PMC9467987; doi:10.1038/s41467-022-33098-y)
Supplement: Supplementary file 3 — Reporting Summary [file 41467_2022_33098_MOESM3_ESM.pdf]

nature portfolio

Corresponding author(s): Yu Zhang

Last updated by author(s): Aug 26, 2022

Reporting Summary

Nature Portfolio wishes to improve the reproducibility of the work that we publish. This form provides structure for consistency and transparency in reporting. For further information on Nature Portfolio policies, see our Editorial Policies and the Editorial Policy Checklist.

Statistics

For all statistical analyses, confirm that the following items are present in the figure legend, table legend, main text, or Methods section.

n/a	Confirmed	
☐	☒	The exact sample size (*n*) for each experimental group/condition, given as a discrete number and unit of measurement
☐	☒	A statement on whether measurements were taken from distinct samples or whether the same sample was measured repeatedly
☒	☐	The statistical test(s) used AND whether they are one- or two-sided<br>*Only common tests should be described solely by name; describe more complex techniques in the Methods section.*
☒	☐	A description of all covariates tested
☒	☐	A description of any assumptions or corrections, such as tests of normality and adjustment for multiple comparisons
☐	☒	A full description of the statistical parameters including central tendency (e.g. means) or other basic estimates (e.g. regression coefficient) AND variation (e.g. standard deviation) or associated estimates of uncertainty (e.g. confidence intervals)
☒	☐	For null hypothesis testing, the test statistic (e.g. *F*, *t*, *r*) with confidence intervals, effect sizes, degrees of freedom and *P* value noted<br>*Give P values as exact values whenever suitable.*
☒	☐	For Bayesian analysis, information on the choice of priors and Markov chain Monte Carlo settings
☒	☐	For hierarchical and complex designs, identification of the appropriate level for tests and full reporting of outcomes
☒	☐	Estimates of effect sizes (e.g. Cohen's *d*, Pearson's *r*), indicating how they were calculated

Our web collection on statistics for biologists contains articles on many of the points above.

Software and code

Policy information about availability of computer code

Data collection	UV Probe Software v2.42, Tecan i-control Software v1.6.19.2, Malvern Zetasizer Software v7.12, FluorEssence Software v3.8, Bruker Xenon Software v1.2, CHI-760E electrochemical software v20.04, WiRE™ Software v2.0, Thermo Avantage Software v5.967, ICP Expert II Software, OMNIC Software v9.6, DIFFRAC plus XRD Commander Software v2.6.1.
Data analysis	Data was analyzed and plotted using Origin 2018 and Graphpas prism 8. HRTEM images were analyzed in DigitalMicrograph 3.4.

For manuscripts utilizing custom algorithms or software that are central to the research but not yet described in published literature, software must be made available to editors and reviewers. We strongly encourage code deposition in a community repository (e.g. GitHub). See the Nature Portfolio guidelines for submitting code & software for further information.

Data

Policy information about availability of data

All manuscripts must include a data availability statement. This statement should provide the following information, where applicable:

- Accession codes, unique identifiers, or web links for publicly available datasets
- A description of any restrictions on data availability
- For clinical datasets or third party data, please ensure that the statement adheres to our policy

All data supporting the findings of this study are available within the article and its supplementary information files. Source data are provided with this paper. Data is available from the corresponding author upon request.

Field-specific reporting

Please select the one below that is the best fit for your research. If you are not sure, read the appropriate sections before making your selection.

- [x] Life sciences
- [ ] Behavioural & social sciences
- [ ] Ecological, evolutionary & environmental sciences

For a reference copy of the document with all sections, see nature.com/documents/nr-reporting-summary-flat.pdf

Life sciences study design

All studies must disclose on these points even when the disclosure is negative.

Sample size	The samping size of the nanoparticles in this study was determined based on the fact that the dection signal of the substances to be measured cannot exceed the maximum detection limit of the instrument in a certain period of time. Nanoparticles were randomly selected from the mother sample for the experiment. The experiment is repeated three times if necessary,and error bars are given in the figures.
Data exclusions	No data was excluded from the analysis.
Replication	Experiments were repeated a minimum of one time to confirm the repeatability of the findings. All repeats of experiments were successful.
Randomization	The samples of the nanozyme solution were prepared in advance as a mother liquor of known concentration. Sonicate for one minute before use to mix the samples well. The appropriate amount was randomly taken as the experimental or control groups.
Blinding	Blinding was not performed in this study because no clinically relevant trials were involved.

Reporting for specific materials, systems and methods

We require information from authors about some types of materials, experimental systems and methods used in many studies. Here, indicate whether each material, system or method listed is relevant to your study. If you are not sure if a list item applies to your research, read the appropriate section before selecting a response.

Materials & experimental systems

n/a	Involved in the study	
x		Antibodies
x		Eukaryotic cell lines
x		Palaeontology and archaeology
x		Animals and other organisms
x		Human research participants
x		Clinical data
x		Dual use research of concern

Methods

n/a	Involved in the study	
x		ChIP-seq
x		Flow cytometry
x		MRI-based neuroimaging
